# Strengthening nutrition policy and service delivery: Lessons learned from a six‐country assessment of Alive and Thrive's technical assistance

**DOI:** 10.1111/mcn.13711

**Published:** 2024-10-03

**Authors:** Kendra Siekmans, Sujata Bose, Jessica Escobar‐DeMarco, Edward A. Frongillo

**Affiliations:** ^1^ Independent Consultant Guelph Ontario Canada; ^2^ Alive & Thrive, FHI 360 Washington DC USA; ^3^ Department of Epidemiology and Community Health University of North Carolina at Charlotte Charlotte North Carolina USA; ^4^ Department of Health Promotion, Education, and Behavior University of South Carolina Columbia South Carolina USA

**Keywords:** capacity building, decision‐making, health services, maternal nutrition, nutrition policy, strategic use of data, technical assistance

## Abstract

Alive & Thrive (A&T) provides strategic technical assistance (TA) to develop effective policies; improve maternal, infant, and young child nutrition (MIYCN) programme design and implementation and enhance system capacity to sustain quality MIYCN service delivery at scale. A qualitative assessment was conducted using document review and stakeholder interviews (*n* = 79) to describe a selection of A&T's TA in six countries and systematically assess the contextual and TA process‐related factors that influenced the results achieved and document the lessons learned about MIYCN TA design and implementation. To facilitate the selection of different types of TA, we classified TA into two levels of stakeholder engagement and intensity. Under the Technical Advisor TA category, we assessed A&T's support to strengthen national policy formulation, monitoring, and implementation of the International Code of Marketing of Breast‐milk Substitutes. For Capacity Development TA, we assessed A&T support to scale‐up maternal nutrition services and to increase strategic use of data. Factors important for TA provision included identifying and engaging with the right people, using evidence to support advocacy and decision‐making, using multiple ways to strengthen capacity, developing packages of tools to support programme scale‐up, and reinforcing feedback mechanisms to improve service provision and data quality. Challenges included shifts in the political context, poorly functioning health systems, and limited resources to replicate or sustain the progress made. Continued investment in evidence‐based and practical TA that strengthens the institutionalization of nutrition across all stakeholders—including government, medical associations, civil society and development partners—is essential. Future TA must support governments to strengthen system capacity for nutrition, including financial and human resource gaps that hamper full scale‐up.

## Key messages

- TA activities helped bridge the gap between enabling nutrition policy environments and programme implementation, supporting governments and partners to institutionalize and operationalize national policies.
- Involving a diverse group of stakeholders in the TA process helped build a common agenda with all who have a role to play but do not traditionally work together.
- Implementation research provided packages of field‐tested nutrition interventions and tools that supported governments in the adoption and scale‐up process.
- Despite well‐designed TA and strong government engagement, barriers to scale‐up were shortfalls in financial and human resources for nutrition activities.

## Graphical abstract

A qualitative assessment describes the nutrition technical assistance provided by Alive & Thrive in six countries, based on desk review and 79 stakeholder interviews. Factors important for TA included engaging with the right people, using local evidence, strengthening capacity, developing packages of field‐tested tools and reinforcing feedback mechanisms.

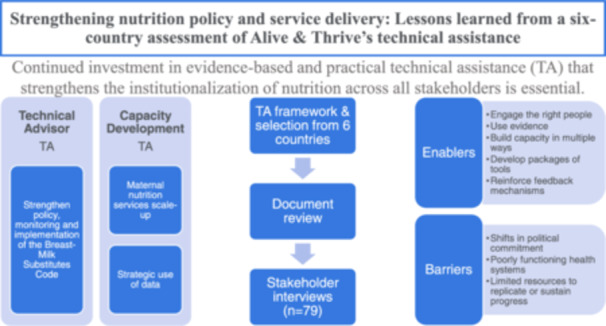

## 1 INTRODUCTION

The global burden of malnutrition among women and children remains unacceptably high (Victora et al., 2021). Despite evidence on the importance of intervening in the first 1000 days and ensuring adequate maternal nutrition, the scale‐up of implementation of nutrition interventions in low‐ and middle‐income countries (LMICs) has been slow, and sparse coverage data show modest gains over the past decade (Heidkamp et al., 2021). The recent impact of multiple shocks—including the COVID‐19 pandemic, conflict, climate change and economic instability—has challenged even the most resilient health systems.

Alive & Thrive (A&T) is a global initiative to save lives, prevent illness, and ensure healthy growth and development through improved breastfeeding, complementary feeding practices and maternal nutrition and health behaviours. After its first phase of demonstrating the potential to improve infant and young child feeding (IYCF) practices at scale, A&T adopted a strategic technical assistance (TA) role in its second phase (starting between 2014 and 2017, depending on the country), supporting those in implementing roles to develop effective policies; improve maternal, infant, and young child nutrition (MIYCN) programme designing and implementation and enhancing system capacity to sustain quality MIYCN service delivery at scale.

TA has been used extensively over the past two decades to assist governments and their development partners to strengthen nutrition policies and programmes (Brown et al., 2020; USAID, 2007). TA is broadly defined as knowledge‐based support that is provided to a government or its development partners with the aim of shaping effective policies and institutions, strengthening implementation and building capacity (Cox & Norrington‐Davies, 2019; Nastase et al., 2020b). Since TA is often provided in complex political and institutional contexts, its design and delivery modality influence its effectiveness, including the role played by the TA provider (Nastase et al., 2020b).

Experience with nutrition‐focussed TA provided through different modalities has been documented, and the results of these efforts assessed in direct or indirect ways (Brown et al., 2021, 2020; Nutrition International, 2020; Siekmans, 2021; SPRING, 2017; USAID, 2007; WFP, 2017). There is a need to build the knowledge base on what works within and across countries, including what modalities can be effective and how the desired results have been achieved (Cox & Norrington‐Davies, 2019).

The effectiveness of A&T interventions in these countries has been evaluated using rigorous methods, and that body of evidence exists elsewhere. Rooted in implementation science (Warren et al., 2020), this assessment seeks to complement those evaluative studies by examining A&T's approach to providing strategic TA governments and partners for MIYCN in Bangladesh, Burkina Faso, Ethiopia, India, Nigeria and Vietnam. We aimed to systematically assess the contextual and TA process‐related factors that influenced the results achieved and document the lessons learned from various stakeholder perspectives that can inform future MIYCN TA design and implementation.

## 2 METHODS

The assessment used a retrospective qualitative study design, including document review and key informant interviews.

### 2.1 Selection of TA activities

**Figure 1:**
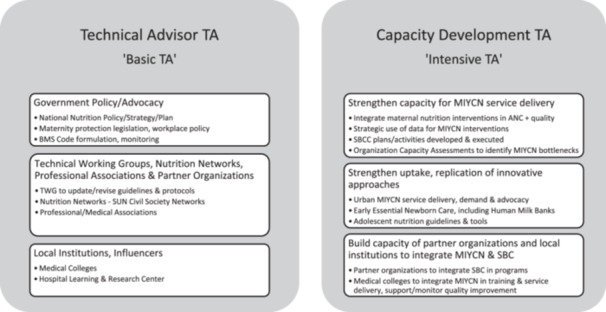
TA classification showing examples of TA activities within each category.

A&T staff in each country provided a list of all current and completed activities that they considered to be TA, including the TA objectives, activities, recipient, provider and dates (TBC see Supporting Information S1: for the full list by country and type of TA)*.* To facilitate the selection of different types of TA, we developed a framework that classified TA by two levels of stakeholder engagement and intensity (Blase, 2009). The first we labelled as Technical Advisor TA, described as TA that facilitates change by providing information/support at a higher or more centralized level, such as the development of policy or programme guidelines, where A&T personnel functioned primarily as expert advisors and knowledge partners to government and partner organizations. The second we labelled as Capacity Development TA, where TA activities had a focus on health system strengthening for nutrition and capacity building at multiple levels (including the service delivery level) for the new knowledge and skills required to achieve change. While these categories were not always mutually exclusive, they served as a basis to organize the analysis. See Figure 1 for examples.

A criteria‐based purposive sample of TA activities was selected for in‐depth review (Gentles et al., 2015), using the list of TA activities from each country as the sampling frame, classified by the two types of TA. One Technical Advisor and one Capacity Development TA activity per country were selected, with preference given to TA activities where the TA process and/or products had been documented, activities took place within the past 2 years (2020–2022) to enhance stakeholder recall accuracy, and a similar type of TA was delivered in at least two countries to compare modalities and outcomes across different contexts. TA activities were ineligible if they were directly covered by other A&T portfolio‐wide learning assessments focussed on social and behaviour change (Flax et al., 2023), maternity protection policy reform (Anderson et al., submitted), and quality assurance/improvement in service provision (Sethuraman et al., 2024).

### 2.2 Data collection

An in‐depth document review was conducted to gather as much information as possible on each TA activity. Information sources included quarterly reports, formative and evaluative research reports, TA deliverables, presentation slides and peer‐reviewed publications.

Key informant interviews with country stakeholders were used to gather information on the experience of implementing or benefiting from TA. Key informants were selected based on direct involvement in the TA, including A&T staff, government stakeholders at national and subnational levels, and others such as development partner organization staff, TA consultants, and donor representatives. The lead author worked closely with A&T staff to identify stakeholders that met the inclusion criteria and invite them by email to participate in a virtual interview.

A semi‐structured interview guide was developed, pilot‐tested and adapted for different types of TA and stakeholders. The guide asked about their role in the TA, the context and TA activities carried out, results achieved (including actions taken post‐TA), lessons learned about the design and delivery of TA, and the unique added value A&T contributes. Questions for A&T staff also asked about the definition of TA in their context, experience with consultant TA providers, and quality assurance actions taken.

A total of 79 individuals (51% women) participated in interviews, ranging from 10 to 15 stakeholders per country (see Supporting Information S1: for a summary of key informants by country). In all countries, over half of the stakeholders interviewed were government staff, partner organization staff (e.g., United Nations agency or civil society organization), donor representatives, or consultant TA providers. A&T staff at headquarters (*n* = 2) and regional level (*n* = 3) were also interviewed.

Interviews were 45–60 min in duration, conducted between March and August 2022 using an online video conferencing platform or by phone, depending on the respondent's preference. Most interviews were conducted in English, recorded (with permission) and transcribed. A bilingual interviewer assisted with French interviews in Burkina Faso and one translated interview in Vietnam. French language interviews were transcribed in French and then translated into English for analysis (using DeepL software).

### 2.3 Data analysis

A summary of each TA activity gave the background and A&T's role, along with a list of primary activities, key results achieved, challenges experienced, and priority next steps required. TA activities were categorized as ‘pull TA’ when provided in response to a request for support or ‘push TA’ when proactively offered to help programmes integrate new knowledge and practices (West et al., 2012). TA results were summarized both in terms of completed deliverables (e.g., policy, guidelines and training modules) and stakeholder‐reported changes in the knowledge and behaviour of TA beneficiaries. Thematic analysis was used to analyse the interview transcripts, using an inductive approach (Braun & Clarke, 2006). Dedoose (Version 7.0.23, 2016) was used for coding and analysis. Emerging themes for individual TA activities were compared and aligned with those within the same type of TA.

### 2.4 Ethics statement

FHI 360's Institutional Review Board (IRB) determined that the study was not human subjects research. Ethical approval was obtained from the University of Dhaka's Institute of Health Economics IRB in Bangladesh, the IRB of the Institut de Recherche en Sciences de la Santé in Burkina Faso, and the Health Research Ethics Committee (HREC) of the Lagos State University Teaching Hospital (LASUTH) in Nigeria. The other countries did not require IRB approval. For Bangladesh, Burkina Faso and Nigeria, signed consent forms were obtained. Although not required, for participants from other countries and the A&T Washington and regional offices, verbal consent to participate and be recorded was obtained.

## 3 RESULTS

### 3.1 A&T's approach to TA

A&T's approach to prioritization and initiation of TA activities varied across countries, but, in general, there was a mix between ‘push TA’, where the initiative came from A&T (or the donor), and ‘pull TA’, where the request for TA came from the government (or development partner). Often A&T would approach governments to integrate emergent evidence on MIYCN into policy and programmes, first generating evidence on context‐specific gaps and then working closely with local stakeholders to identify the best ways of filling those gaps. For example, in Burkina Faso, the WHO release of new antenatal care guidelines in 2016 created a demand for TA among the national maternal health programme staff to support them in operationalizing the guidelines, building on A&T's previous experience and tools developed during the integration of maternal nutrition in ANC in Bangladesh. Where A&T had long‐term funding, an increase in demand from governments and development partners for ‘pull TA’ was observed as MIYCN was given higher priority, and A&T's role as a technical advisor and knowledge partner was better understood.

A&T staff provided the majority of TA, only contracting external consultants for specific components. Each country positioned their staff in different ways: embedded senior technical advisors in government offices at national and subnational levels (Burkina Faso and Ethiopia), subnational teams to work with government and development partners on TA and other activities (Nigeria and India) and a regional pool of technical advisors to support MIYCN TA across several countries (Vietnam).

A&T staff who provided TA were described as having strong technical expertise, an ability to draw on both global and local evidence for what works to improve MIYCN and a solid understanding of local health systems and stakeholders. Most were medical doctors or senior health professionals with PhDs and many years of experience working in the country's health system. A&T staff also had access to a global network of colleagues and subject matter experts who supported the TA delivery process.

### 3.2 Technical Advisor TA

The Technical Advisor TA activities assessed sought to strengthen the national policy formulation, monitoring, and implementation of the International Code of Marketing of Breast‐milk Substitutes (the Code) (see summary in Table 1) (see Supporting Information S1 for detail). In Bangladesh and India, we assessed A&T's TA activities to enhance awareness and adherence to the Code among professional medical association members, building on existing partnerships with these associations. In Vietnam, we assessed A&T TA to strengthen the engagement and capacity of the SUN Civil Society Alliance members in Code monitoring, working with government health inspectors to co‐design and co‐facilitate a training workshop. In Burkina Faso, Ethiopia, and Nigeria, A&T TA supported government‐led Code policy, implementation, and monitoring efforts. In Burkina Faso and Ethiopia, A&T collaborated with UNICEF to provide TA to strengthen Code legislation, as well as support dissemination and sensitization activities in Ethiopia. In Nigeria, A&T supported the government's development of a 5‐year strategic plan for implementation and monitoring of the Code, working with national and international code experts, as well as UNICEF.

**Table 1:** Description of Technical Advisor TA activities by country.

Country and TA partner	TA provider and timeframe	TA activity description	TA results	TA challenges
**Bangladesh** Professional Medical Associations	A&T Staff 2017–2022	Letter of Collaboration (LOC) with Ob‐Gyn Society Bangladesh (OGSB) and Bangladesh Paediatric Association (BPA) to provide TA to uphold adherence to BMS Act; highly influential advocacy from esteemed medical associations.	OGSB/BPA‐endorsed BMS Act briefs for three target audiences that were co‐developed with IPHN. Increased engagement by government in advocacy & dissemination of BMS Act assessment results. Increased awareness among association members of the BMS Act and their responsibilities to uphold it.	Delays in official endorsement of the briefs. Lack of funds for implementation requires A&T to depend on government and other partner organizations to mobilize resources.
**India** Professional Medical Associations	A&T Staff 2017–2022	Letter of Collaboration (LOC) with Indian Academy of Paediatrics (IAP), Federation of Gynaecologists and Obstetricians in India (FOGSI), and Indian Association of Preventive and Social Medicine (IAPSM) for adopting a strong position on MIYCN, develop and implement evidence‐based MIYCN guidelines, and enhance awareness and adherence to IMS Act.	Joint development by FOGSI, IAP and IAPSM of MIYCN policy & guidelines, integration of MIYCN in pre‐service and in‐service training, assessment of health service provider knowledge and practices. IMS Act content included in medical college curriculum and CME programmes. Identification of MIYCN champions within each association and formation of MIYCN working groups.	Time required to identify the appropriate subgroup within each association to work on IYCF and maternal nutrition issues. Limited political will of government ministries for IMS Act implementation and monitoring.
**Vietnam** SUN Civil Society Alliance (CSA)	A&T Staff 2020	Capacity building to SUN CSA on Code monitoring and reporting on violations and violators.	Code violation training workshop. Increased awareness and engagement by SUN CSA member organizations in detecting Code violations and related issues (e.g., workplace lactation policy). Code compliance prioritized in SUN CSA workplan for 2021–2023. Increased CSA member and government capacity to respond to infant formula safety issue, support to health safety inspectors.	Legal loopholes used by companies limit the government's ability to act on the reported Code violations.
**Burkina Faso** Government MDA	A&T Staff, Consultant 2018–2022	Technical support to update and strengthen the BMS Code decree through roadmap preparation, legal advice, technical workshops with BMS Code allies, and surveillance of BMS Code violations using NetCode protocol.	Adoption and signing of the new Code decree by all relevant ministries. MOH commitment to scale‐up IYCF, revise hospital standards of care, and accelerate BFHI implementation. Complemented with Stronger with Breastmilk Only campaign, TA has contributed to increase in breastfeeding and less visible violations by companies and manufacturers.	Large number of stakeholders involved required many meetings to resolve issues raised by different parties. Hiring government lawyer to facilitate legal process was effective but resulted in longer than expected timelines due to other demands on his time.
**Ethiopia** Government MDA	A&T Staff 2018–2022	Technical support for adaptation of BMS Code and sensitization/dissemination for national and regional stakeholders.	BMS Directive revised, officially adopted, and translated into Amharic. Code‐related materials and tools — one‐page brief, advocacy briefs for target groups, posters — produced, translated into local languages and distributed at national and regional levels. Increased capacity to monitor Code violations using health facility inspection checklist which now includes 10 violation‐related indicators.	Planned assessment of Code violations in urban areas was cancelled due to the civil war. Frequent turnover in government staff. Limited reach of training and sensitization activities to field‐level health workers and private sector health care providers.
**Nigeria** Government MDA	A&T Staff, Consultant 2018–2022	Technical support to develop and launch NAFDAC's 5‐year strategic plan to improve Code implementation, monitoring and compliance.	NAFDAC's 5‐year costed strategy was launched in August 2021. Increased capacity for planning and resource mobilization using costing information. A&T and UNICEF's support has contributed to strengthening NAFDAC's enforcement of the Code through having a better articulated operational and policy framework (national regulations), an active Technical Committee on the Code and awareness creation efforts.	Changes in NAFDAC leadership caused delays and required renewed advocacy efforts by A&T. Efforts to cost the strategy were delayed.

Across A&T's Code‐related TA activities in these six countries, several common themes emerged about factors that contributed to the TA results achieved. The first theme was the importance of identifying and building strategic partnerships with key stakeholders who needed to be involved in the TA process. Key informants spoke about A&T's role in bringing them together at the outset to discuss and build consensus on what to do, what role each stakeholder could play, and how to work together. In Bangladesh and India, A&T built strategic alliances with professional medical associations who are influential, both in society generally and more specifically with government officials and health care providers in both private and public health settings. This enabled A&T to increase awareness of the Code and the responsibility to uphold it among a wide range of medical professionals. It also contributed to achieving several institutional‐level results, including joint development and adoption of evidence‐based guidelines to improve MIYCN knowledge and practices and integration of MIYCN in pre‐service and in‐service training.

Evidence also showed the value of involving different stakeholders in the TA process to help build a common agenda with all who have a role to play but do not traditionally work closely together. For professional medical associations, this meant engaging medical professionals across the continuum of prenatal to postnatal care to achieve consistent infant feeding guidelines for both obstetricians and paediatricians.

[we brought] together IAP, FOGSI and IAPSM … we got them to do a joint guidance statement on [supporting] early initiation of breastfeeding during C‐section. …if your obstetrician is not ready to support that, the neonatologists and paediatrician comes into the picture much later. And prior to that, you have already the formula milk prescribed. (A&T staff, India)

In Burkina Faso, engaging all relevant government ministries, departments, and agencies resulted in all signing the new Code decree. In Vietnam, A&T's TA process supported ‘partnership building and engaging civil society and the government in a constructive policy dialogue’ (A&T Staff, Vietnam). Building alliances of stakeholders with common interests was also useful for leveraging partner organizations' technical expertise and resources to achieve more than what A&T itself could support.

A second common theme was A&T's use of evidence and previous experience in MIYCN implementation research and programmes to convince decision‐makers. Assessments of Code compliance (using NetCode protocol) during the TA process raised awareness of local Code violations and provided credible evidence for the government and medical professionals to act upon. A&T also contributed to building the capacity of the government and its partners to conduct these assessments; however, efforts to sustain these Code monitoring efforts were in the early stages in most contexts.

Third, Code‐related TA required extensive knowledge transfer activities to support changes in stakeholder awareness and behaviours. Using A&T's in‐house communication expertise, A&T TA providers gave technical support to disseminate information on the Code to key stakeholders within and outside the health system through events (e.g., World Breastfeeding Week) and trainings (e.g., baby‐friendly hospital initiative, continuing medical education courses). Although A&T helped organize them, events often were led by the government or medical associations themselves, building capacity for communicating the information and reinforcing their responsibility for this lead role.

A&T invites people from the Ministry of Health, who are in charge of monitoring the BMS Code to conduct the training. … It's very good that, one hand, the Ministry of Health can provide information for us, but I think another more important, that it emphasizes their roles to do that.… that they are the person who has the responsibility. (Partner organization)

Challenges experienced by Code‐related TA activities included unexpected shifts in the political context that limited achievement of desired outcomes and sustainability, despite using approaches that fostered strong government engagement and ownership. Nutrition alliances in each context faced barriers such as frequent government personnel changes, a lack of political will to engage on Code‐related issues, and limited government resources to replicate or sustain the progress made. Government stakeholders, in particular, expressed a need for continued technical and financial support by A&T and other development partners to complete processes that were still in the early stage of implementation.

In this strategy, there are budgets and there are proposals for so many things, like the institutionalization of the Code. … There are supposed to be meetings, reviews, validations…but because of funding, we cannot do a lot of these things. (State‐level government stakeholder, Nigeria)

A summary of A&T staff and other stakeholder reflections on the lessons learned during these TA activities is shown in Table 2.

**Table 2:** Summary of lessons learned by TA type from stakeholder reflections.

**Technical Advisor TA** Building trust and good partnerships, even when it takes patience and time Finding (or making) the champions who can be influential and effective agents for change Taking time to understand the political situation and the field realities that people face Thinking outside the box—innovative approaches to TA and partner engagement Continuing support to processes that are still in early stages of development
**Capacity Development TA** Taking into consideration the broader context for health and nutrition services, including health system functionality, when seeking to strengthen nutrition within existing health service delivery platforms Capacity strengthening as a process requiring an institutionalized approach that goes beyond training workshops for health workers An ability to adapt to unexpected changes in implementation context Political will by governments to scale‐up effective interventions Importance of TA provider stature (seniority, technical expertise) and credibility to be effective in their role

### 3.3 Capacity Development TA activities

The sample of A&T activities selected under the Capacity Development TA category focused on support to the scale‐up of maternal nutrition services within the health system (*n* = 3) and building government capacity for strategic use of data (*n* = 3). See summary in Table 3 (see Supporting Information S1 for details).

**Table 3:** Description of Capacity Development TA activities by focus area and country.

Country and TA partner	TA provider and timeframe	TA objective and main activities	TA results	TA challenges
**Maternal Nutrition**				
**Bangladesh** Civil Society Organizations (CSO), National Nutrition Services, Institute of Public Health and Nutrition	A&T staff 2019–2022	**Objective**: To build capacity of two CSOs to strengthen MICYN service delivery in urban facilities and generate demand for those services **TA Activities**: • Develop urban MIYCN counselling implementation guide & materials • Train CSO urban health facility project managers, MIYCN counsellors and Community Workers how to implement MIYCN counselling model • Technical support, training and mentoring for quality implementation • COVID‐19 adaptation: remote training, supervision, mobile‐MIYCN • Advocacy with government and partners to include this model in urban strategy and project design	Urban MIYCN counselling programme package Increased capacity of CSO staff to provide quality MICYN counselling services Increased community demand for nutrition counselling services CSO commitment to sustain model with cost‐recovery approach Government commitment to adopt and scale‐up model, including set‐up of counselling room in new IPHN building Evidence generated on feasibility and effectiveness of the model (pending publication of evaluation findings)	Inability to conduct research using government platform due to contractual issues COVID‐19 pandemic onset during first week of training required multiple adaptations to approach and limited dissemination of baseline findings to government and other stakeholders High levels of facility staff turnover
**Burkina Faso** Ministry of Health—Directorate of Family Health (DSF) and Directorate of Nutrition (DN)	A&T staff June–November 2021	**Objective**: To integrate maternal nutrition interventions in maternal health services at scale following results of the implementation research. **TA Activities**: • Support DSF/DN to disseminate results of implementation research • Support MOH to revise and validate maternal nutrition training modules, national SBC strategy and package of communication tools for various levels • Support MOH to develop national scale‐up plan based on lessons learned and minimum package of maternal nutrition interventions	MOH updated and validated maternal nutrition training modules Scale‐up of minimum package of maternal nutrition interventions across all health facilities in four districts where study was done Integration of maternal nutrition in regional reproductive health supervision checklist Integration of MOH standards on quality of maternal, newborn care Enhanced capacity of health workers to monitor weight gain during pregnancy	High cost for government to print job aids and SBCC materials (limits scale‐up of these tools) Lack of coordination with other development partners to use MOH‐endorsed maternal nutrition training modules
**India** Ministry of Health and Family Welfare, Uttar Pradesh (UP) State Health Mission, UP‐Technical Support Unit	A&T staff 2018–2022	**Objective**: To provide technical support for government and partner organizations in UP, Bihar and Jharkhand states to integrate and strengthen maternal nutrition interventions during ANC, and national‐level technical support to MoHFW to strengthen the institutional enabling environment for maternal nutrition. **TA Activities**: • Integrate/strengthen maternal nutrition in ANC platform in 2 UP districts • Advocacy with MOHFW/state Health Mission of UP and partner orgs • Share learning from implementation research at state and national levels • Provide content input to national guidelines on integrating MN during ANC service provision and SBC toolkit for dissemination across states • Develop system strengthening package with tools for adaptation/adoption by government and partners • Work with Maternal Nutrition Partners Consortium on technical webinars	Increased recognition by government and development partners that maternal nutrition is a priority for action. Strengthened health system in two UP districts where the IR was conducted Strengthened collaboration with UP state government and partners (UP‐TSU) based on practical tools and learning supported by A&T Increased capacity (technical knowledge, training materials, budget) of UP‐TSU to support government to train frontline workers on maternal nutrition ICDS commitment to scale‐up mobile phone applications for supportive supervision and strategic use of data components across all 75 UP districts Inclusion of maternal nutrition training in state PIPs, resulting in increased funding available for nutrition actions and increased motivation to implement these.	COVID‐related impact on results dissemination activities Need to establish new government mechanisms for planning and budgeting of maternal nutrition actions Shifts in political leadership and delays in finalizing the revised national ANC guidelines Need for health system strengthening as well as technical nutrition intervention delivery
**Strategic Use of Data**				
**Ethiopia** Regional Health Bureaus, Federal Ministry of Health	A&T staff, consultant 2019–2022	**Objective**: To improve nutrition (MIYCN) data use at federal and regional levels. **TA Activities**: • Training for regional M&E and nutrition programme officers • Nutrition Information System Assessment, results dissemination and prioritization of TA support • Revitalize RHB performance management teams to improve data use • Support to include nutrition indicators in Routine Data Quality Assessment	Report on Landscape Analysis of Nutrition Information Systems in Ethiopia (Telos Consulting, 2020) Revitalized performance management team meetings in the RMNCH directorate in three regions Enhanced capacity of nutrition case teams to conduct weekly, monthly and quarterly reviews of data reported in DHIS2 and provide feedback to lower levels on data quality and performance Integration of nutrition in health sector tools (e.g., nutrition indicators in supportive supervision checklist) and information systems content in nutrition implementation guide and training materials. (1 RHB)	Delay in assessment report dissemination due to COVID‐19 pandemic restrictions Disruption in TA for two regions due to conflict Although the project sought to strengthen multi‐sectoral nutrition programming, resource constraints required A&T to narrow the scope of nutrition information system work to the health sector Large regions require large‐scale efforts that go beyond what government or A&T can support
**Nigeria** Kaduna State Primary Healthcare Development Agency	A&T staff 2020	**Objective**: To build capacity and develop a monitoring framework for use in monitoring and mentoring of IYCF data capture and quality **TA Activities**: • Ensure all 2019 NHMIS tools are available and in use, especially the GMP register • Conduct joint supportive supervision (data quality assurance) visits with LGA M&E officers to PHCs and private health facilities to build capacity of staff in data validation and troubleshooting data errors • Develop a monitoring and mentoring framework • Participate in state government data review meetings	Enhanced knowledge and skills of state and LGA‐level health workers to use 2019 NHMIS tools, including electronic data entry to DHIS2 using mobile phones Increased availability of data tools (registers, summary forms) at health facilities Increased quality of data reported—timeliness, high reporting rate (80%–90%), fewer data gaps, improved internal consistency Integration of nutrition indicators in routine monthly and quarterly data quality review meetings	High rate of staff turnover, staff transfers that resulted in loss of trained personnel COVID‐related restrictions resulted in low, incomplete data documentation and reporting Government staff reliance on A&T technical support (and presence) to implement monitoring framework Mobile app dependent on funding for network access and regular maintenance of phones
**Vietnam** Maternal and Child Health Department, Ministry of Health	A&T staff 2021	**Objective**: To develop checklists for facility assessment, questionnaires and platform for mothers' experience survey for the Center of Excellence for Breastfeeding initiative **TA Activities**: • Work with MOH to develop COE designation, evaluation criteria and designation process • Develop tools and materials to support hospitals to meet the COE criteria (in collaboration with MCH department and Da Nang LRC), including tools to assess and monitor progress (communication products, coaching material, tools, checklists) • Provide TA to hospitals to improve breastfeeding support practices with Da Nang LRC as master trainers • Develop mother experience survey to provide feedback to hospitals on their performance; build capacity of CDC/DOH/MOH to conduct surveys on quarterly basis as independent assessment • Support COE hospitals to identify gaps and develop action plans for improvement. Provide expert observation, coaching and supportive supervision to enable hospitals to reach the benchmarks • Support MOH to create a monitoring process and tool for COE‐designated hospitals to continue conducting mother experience surveys on a quarterly basis, as a way to ensure they remain COE compliant	In 2022, 70 hospitals across 14 provinces are enroled in the COE initiative (up from 28 hospitals in 8 provinces in 2019). 23 hospitals are officially designated as COE and 10 are waiting a decision. In Q3 of 2022, 15 out of 23 COE hospitals conducted the quarterly survey; others supported by provincial DOH/CDC MOH adopted Decision No. 3451 (2019) approving the COE designation criteria and mechanism in obstetric hospitals; updated in 2021 (decision 5913/QD‐BYT) to allow paediatric hospitals to participate in the initiative Enhanced capacity of MOH/DOH and hospitals to obtain regular feedback on hospital EENC practices, with quarterly monitoring surveys of mothers' experience providing timely data for action Enhanced knowledge and skills of hospital staff, resulting in improved EENC practices and labour/delivery practices which in turn contribute to better patient outcomes and cost savings for the hospitals (Omer‐Salim, 2020) Increased early and exclusive breastfeeding practices reported by women who delivered in COE‐enroled (Joyce et al., 2021) and designated hospitals (Omer‐Salim, 2020)	COVID‐19 pandemic shifted priorities for hospitals and delayed scale‐up process Low commitment of hospital leadership in small number of hospitals Variable level of capacity of hospitals and DOH to conduct quarterly monitoring surveys

#### 3.3.1 Support to scale‐up of maternal nutrition services

In Burkina Faso and India, A&T supported the government at national and subnational levels to integrate and scale‐up maternal nutrition in the health system, utilizing training materials, tools and learning from maternal nutrition implementation research studies that A&T had completed (Kim et al., 2023; Nguyen et al., 2021). In Bangladesh, A&T provided TA to two civil society organizations (CSOs) for the delivery of nutrition interventions during ANC in an urban context as part of an implementation research study, which was on‐going at the time of this assessment due to COVID‐19 pandemic‐related delays (Nguyen et al., 2023). Throughout the TA process, A&T engaged with, and advocated for, the government to adopt the approach.

Several factors emerged as important for maternal nutrition TA provision. In all three contexts, TA for scale‐up benefited greatly from the learning and insights gained during the implementation research that preceded it. The evidence generated reinforced A&T's credibility as a knowledge partner for the government and increased national and subnational‐level decision‐makers' engagement. In particular, government personnel working in the health system appreciated the knowledge generated on what works to improve health worker skills and practices, increase client demand for maternal health and nutrition services, and increase awareness of the importance of nutrition during the antenatal period and early childhood.

Strengthening the nutritional service is a necessity, is important for mother and child nutrition. It is known and accepted fact by all the stakeholders in Bangladesh. No legislation is needed there. But it was not known how do we strengthen this service in our primary healthcare platform. (A&T staff, Bangladesh)

…we also saw how the capacity building or use of job aids helped, or how the frontline supervisors, once trained, how they supported. …when it came to supportive supervision or use of data, it was very clear that we showed them that this is possible. (A&T staff, India)

A&T's support for the production (during the implementation research process) of a package of high‐quality, context‐specific, user‐friendly and field‐tested training materials and job aids was useful during follow‐up TA to support governments and development partners in the adoption and scale‐up of maternal nutrition interventions.

…Alive & Thrive has developed a tool that allows to follow the weight gain of the pregnant woman throughout her pregnancy and…to prevent malnutrition in pregnant women. This tool has been well appreciated and has also been taken into account by the Ministry of Health. (A&T staff, Burkina Faso)

Supportive supervision and strategic use of data featured consistently in A&T TA activities aiming to strengthen nutrition interventions within existing health services. Stakeholders described how these strategies improved both the delivery of maternal nutrition services and health worker practices and motivation generally. The demonstrated value of these strategies during the implementation research process was one driver of interest to scale‐up the package of maternal nutrition interventions among decision‐makers (both government and non‐government, alike) in all three countries assessed.

We have a better… partnership with government, like Women and Child Development, as well as health department because of our learnings and our ideas on supportive supervision and use of data. … They like we are providing a solution to them, and they were happy to partner with us to get the technical support and scale up. (A&T Staff, India)

Common challenges also emerged. A&T maternal nutrition TA activities were designed to support the government and its partners to scale‐up demonstrated models supported by a package of field‐tested tools. Despite A&T's approach in working closely with the government throughout the TA process, it was a challenge for governments to take the evidence forward and scale‐up on its own. Results have been modest to date, with government and development partners looking to A&T for on‐going TA to support the scale‐up process.

For the urban model, now it is the time to scale up and mainstream throughout the health system…. If there is no more advocacy and TA support, then it'll be stopped. Because without this technical support, government will not be able to mainstream and scale up. (A&T staff, Bangladesh)

Also, while maternal nutrition TA activities aimed to strengthen the delivery of nutrition interventions within existing health service delivery platforms, poorly functioning health systems made this difficult to achieve. A&T TA providers worked with government staff to identify bottlenecks that impacted nutrition services and invested time and resources in health system strengthening initiatives alongside the nutrition‐focused components. This increased the time and resources required to achieve their nutrition objectives.

You spend a lot of time to make the system ready. Once that system is ready, then you start working on the specific tool … like maternal nutrition. So it takes time in India … You spend your energy for system readiness, system strengthening, then you come to the technical intervention. (A&T staff, India)

#### 3.3.2 Strategic use of data

TA activities for strategic use of data assessed A&T's support for building government capacity for improving the quality, reporting and use of data on MIYCN interventions. In Ethiopia, A&T conducted a landscape analysis of information systems for nutrition across multiple sectors, consulted with government stakeholders to prioritize gaps that could be filled and worked with the Ministry of Health (MOH) at both federal and regional levels to improve nutrition data quality and use for programme strengthening. In Nigeria, A&T provided technical and financial support in two states to roll‐out revised health management information system (HMIS) tools and training of health workers. Working at state, local government area, and health facility levels, the TA activities sought to strengthen the use of the tools and improve the quality of data for nutrition (specifically IYCF) indicators. In Vietnam, the A&T team provided TA to the government in developing a novel approach to strengthening baby‐friendly care provided by hospitals through the Centers of Excellence (CoE) for Breastfeeding Initiative. The TA activities focus on the strategic use of data, including the development of checklists for the CoE designation criteria and an online monitoring platform for the government and designated hospitals to conduct quarterly mothers’ experience surveys via telephone about the services provided by hospitals.

Themes that emerged as important for the strategic use of data TA provision were as follows. Supportive supervision was a key mechanism for providing on‐going capacity building, reinforcing training received, and mitigating the impact of high staff turnover at health facilities. In Ethiopia and Nigeria, A&T staff modelled how routine supportive supervision was useful to increase awareness of nutrition services and indicators, strengthen the quality of nutrition data collected and used and strengthen coordination among HMIS, nutrition and frontline health staff. TA support to integrate nutrition items in supportive supervision checklists was expected to promote sustained attention to these activities.

…we are giving the feedbacks for the lower levels of the facility, woredas, zone. For each lower level, then they are improving the data on monthly basis. By using that feedback frequently, they improve their data quality, timeliness, and the others. (Government stakeholder, Ethiopia)

In Vietnam, the quarterly mothers' experience survey results provide data on hospital practices that empower both the hospitals and the government to respond in a timely manner to shifts in practices (Joyce et al., 2021; Omer‐Salim, 2020). For example, hospitals were alerted when the survey results showed a drop in skin‐to‐skin contact during the first year of the COVID‐19 pandemic and were able to take action to address the problem.

…[after A&T's initial work,] it now belongs to the Health Department to call every three months to interview the mother. So I think that reflects the progress of the hospital in terms of maintaining the breastfeeding practice, and that always reminds the health professionals to see how they are doing. (Government stakeholder, Vietnam)

Stakeholders in multiple contexts spoke of the need for on‐going capacity strengthening for strategic use of data, a process that requires an institutionalized approach, going beyond one‐off training events. In Ethiopia, A&T embedded technical advisors in federal and regional government offices where they were able to provide coaching and support to problem‐solving by government staff throughout the year. Both the technical advisors and their government counterparts spoke of the value of this approach.

…by getting the Alive & Thrive team in the office,…the RHB [Regional Health Bureau], benefited in many sides, but especially by focus on system strengthening. …By working in the same office, in one compound, everything is communicated at the same time. The right time at the right place. (Government staff, Ethiopia)

Another promising model for building long‐term and continuous access to local expertise in breastfeeding and essential newborn care was evident in Vietnam, where A&T supported a local hospital Learning and Resource Center to provide coaching and TA to other hospitals in the country.

One key challenge faced by TA focussed on nutrition data was finding the right balance between broader health systems strengthening efforts and those more explicitly focussed on nutrition, particularly in low‐resource contexts where needs are great. For example, in Ethiopia, A&T sought to improve the quality and use of nutrition programme data, but this was impossible to fully achieve without addressing larger HMIS issues beyond the scope of A&T's TA.

## 4 DISCUSSION

A&T delivered a wide range of TA spanning the full spectrum from strengthening the enabling policy environment for nutrition to increasing the reach and quality of MIYCN interventions to the population. Although providing TA was a complex and challenging process, A&T provided support at multiple levels and with multiple stakeholders, using approaches that considered the complexity of the health system and the many different areas where change needed to happen. A&T staff functioned effectively as technical advisors and knowledge brokers, often playing the role of ‘linking agents’ who fostered trust and collaborative working relationships with a wide range of stakeholders and convinced them to work together towards a common goal (Glegg & Hoens, 2016; West et al., 2012).

Most of the TA activities assessed achieved the desired outputs. Factors important for TA provision included identifying and engaging with the right people, using evidence to support advocacy and decision‐making, using multiple approaches to strengthen capacity, producing field‐tested packages of training materials and tools to support programme scale‐up, and reinforcing feedback mechanisms to improve service provision and data quality. Despite a different duration of A&T's presence in each country, there was similar commitment across countries to an inclusive, partnership‐building approach to TA. The observed ability of TA providers to build and manage strategic partnerships was likely related to the A&T staff's seniority, technical expertise, and familiarity with the local context.

Challenges to TA process and achievements included shifts in the political context, poorly functioning health systems that limited the extent to which A&T TA could achieve changes and limited government resources to replicate or sustain the progress made during the TA process. All three factors affect the extent to which governments can drive the TA process, the optimal timing of TA provision, and the length of time required to achieve progress. Incorporating political economy analysis and capacity assessment at design stage can help inform the types of activities needed and relevant stakeholders involved (Balarajan & Reich, 2016; Brown et al., 2021).

A&T TA activities often helped bridge the gap between an enabling policy environment and programme implementation. While broad political commitment for nutrition and relevant nutrition policies were already in place, there was a need to convert this *expressed* commitment into *institutional* and *operational* forms of commitment for nutrition (Baker et al., 2018; Fracassi et al., 2020). A&T TA directly contributed to progress by government and development partners in institutionalizing MIYCN policies to enable increased quality and scale of programming. For example, technical support on *how* to implement nutrition guidelines and protocols contributed to adapted and streamlined processes and tools for delivering maternal nutrition interventions as part of ANC (Sanghvi et al., 2022). TA support to integrate nutrition actions in government annual plans and budgets also contributed to building increased operational commitment for nutrition.

The findings show the value for TA of context‐specific evidence from implementation research that supports the design and delivery of interventions (Frongillo & Escobar‐Alegria, 2021; Menon et al., 2014). Demonstrated success in implementation shows government stakeholders the feasibility of integrating nutrition interventions in existing health service delivery platforms (Gillespie et al., 2021). TA based on local evidence is also more convincing for politicians and administrators to allocate resources. Sharing this evidence with national and subnational‐level stakeholders helped to strengthen vertical coherence for nutrition interventions (Gillespie et al., 2015), which is particularly important in countries with decentralized governments, such as Ethiopia, India and Nigeria, where regions or states need to domesticate national nutrition policies and strategies (Adeyemi et al., 2022; Frongillo & Escobar‐Alegria, 2021).

Our results also highlight the importance for TA to build a common agenda for nutrition among groups of stakeholders who may not normally work together. Building alliances was important particularly for TA processes focussed on strengthening nutrition policy (Burkina Faso and Ethiopia), policy monitoring and compliance (Bangladesh, India and Vietnam), and institutional strategy development (Nigeria). While building coalitions across different groups can be a time‐consuming and challenging process, it helps to address the political economy challenge that nutrition often faces as a less organized and mature profession (Balarajan & Reich, 2016). Alliances with influential professional medical associations also strengthen credibility and garner the attention of key decision‐makers. Analysis of power relations between key actors can also influence the scale‐up strategy, such as for childhood obesity in Brazil (Machado et al., 2023).

Capacity strengthening at all levels is essential for improving the quality and scale of nutrition interventions (Nastase et al., 2020a; Shrimpton et al., 2014). A&T's TA included various training activities to build capacity, often using techniques that emphasized practical skills. Stakeholders also appreciated and benefited from strategic and operational capacity development efforts (Gillespie et al., 2015), including coaching by A&T technical advisors embedded in government offices, on‐going access to A&T staff expert input, and collaboration in implementation research. A review of breastfeeding TA in Malawi found it was primarily used for competency building, including training, coaching mentorship, and supervision, but needed to consider other areas, particularly monitoring and data management (Mukuria‐Ashe et al., 2022). Governments and development partners need to formally assess capacity for nutrition and develop long‐term capacity development strategies (SUN Movement, 2021). Others have also identified the need for national nutrition workforce strategies and implementation plans to improve individual, organizational and system capacity for nutrition (Adeyemi et al., 2022).

Both government stakeholders and A&T staff expressed concern about the ability of governments to move forward without continued support from an external TA provider. This finding is consistent with other evaluations raising concerns about the lack of sustainability of the external TA model (Hernández‐Cordero et al., 2022). In two of the TA activities assessed, A&T invested in building local expertise to ensure sustained access by governments and partner organizations to sources of MIYCN TA, including national institutions capable of conducting implementation research (Gillespie et al., 2015).

Despite largely successful TA delivery, two key barriers to increased scale of programme implementation emerged in all countries – the need for increased financial and human resources to deliver these interventions. Government reliance on external funding has been shown to limit scale‐up of nutrition programmes, such as BFHI (Mukuria‐Ashe et al., 2022). Leadership, operational capacity, and finance are key factors for enabling nutrition intervention delivery (Bhutta et al., 2020; Heidkamp et al., 2021). Nutrition‐focused TA needs to explicitly address financing and resource mobilization to support nutrition programme delivery. This may require a different skill set for TA providers to advocate for a strong commitment from policymakers for increased investment of domestic resources in MIYCN (Zagre, 2021). It may also require different modes of TA delivery. TA providers embedded in subnational government offices can be effective in advocating for increased budgets and spending on nutrition activities, given their on‐going presence at key planning and decision‐making meetings (Siekmans, 2021).

### 4.1 Limitations

This assessment did not cover the full breadth of TA that A&T offers, and while the selection criteria enabled comparison of similar types of TA across countries, this selection also limited the diversity of TA activities included. It was difficult at times to identify the start and end points of discrete TA activities during stakeholder interviews, as it was only one component of a larger agenda for change. No TA activities had clearly articulated a results pathway to link their outputs to expected outcome‐level results. This made it difficult to define and compare the effectiveness of TA activities and identify the contribution of the activities to the results observed. Other assessments of nutrition TA have had similar constraints, particularly when TA supports policy and governance (Siekmans, 2021). All interviews and analyses were conducted by one researcher, enhancing consistency. The virtual interview format, language and cultural differences, and under‐representation of some types of stakeholder types due to staff turnover or non‐response, however, likely resulted in some measurement error. While there is also a potential risk of bias associated with only interviewing stakeholders involved in the TA process, bias was minimized by ensuring a broad selection of stakeholder types and triangulation of the information gathered.

## 5 CONCLUSION

Evidence‐based and practical TA was provided to countries seeking to increase the reach and quality of their MIYCN programmes. This assessment has presented evidence on the key strategies, as well as challenges of this work across a subset of TA activities. Continued investment in TA that strengthens the institutionalization of nutrition across a wide range of stakeholders—including government ministries, departments and agencies, professional medical associations, civil society and development partners—is essential. There is also a need for sustained investment in TA that supports taking to scale the learnings from implementation research and ensuring stakeholder access to on‐going capacity building and coaching support in the technical areas in which TA has been provided.

## AUTHOR CONTRIBUTIONS

Kendra Siekmans designed the assessment protocol, performed all data collection and analyses and wrote the paper. Sujata Bose, Jessica Escobar‐DeMarco and Edward A. Frongillo conceptualized the research, provided input on the design, supported the recruitment of participants and preparation for ethical reviews (FHI 360 and countries) and contributed to the manuscript. Kendra Siekmans worked closely with A&T staff to identify stakeholders who met the inclusion criteria and invited them by email to participate in a virtual interview. All authors have read and approved the final manuscript.

## CONFLICT OF INTEREST STATEMENT

The authors declare no conflict of interest. Drs. Escobar‐DeMarco and Bose were members of the A&T assessment team but were not involved in implementation of any of the TA studied for this manuscript.

## Supporting information

Supporting information.

## Data Availability

The data that support the findings of this study are available on request from the corresponding author. The data are not publicly available due to privacy or ethical restrictions.
