# Supplementary material for: Strengthening nutrition policy and service delivery: Lessons learned from a six‐country assessment of Alive and Thrive's technical assistance
Source: Matern Child Nutr. 2024 Oct 3;21(2):e13711. doi: 10.1111/mcn.13711 (PMC11956047; doi:10.1111/mcn.13711)
Supplement: Supplementary file 1 — Supporting information. [file MCN-21-e13711-s001.docx]

**Strengthening nutrition policy and service delivery: lessons learned from a six-country assessment of Alive & Thrive’s technical assistance**

Supporting Information

Contents

[Key Informant characteristics 2](#_Toc120723100)

[Country Summaries of Technical Advisory TA 3](#_Toc120723101)

[Country Summaries of Capacity Development for Maternal Nutrition TA Activities 10](#_Toc120723102)

[Country Summaries of Capacity Development for Strategic Use of Data TA Activities 14](#_Toc120723103)

## Key Informant characteristics

A total of 79 individuals participated in interviews, including 40 women and 39 men. The number of stakeholders consulted in each country ranged from 10 in Ethiopia and Viet Nam, to 12 in Bangladesh and India, 14 in Burkina Faso and 15 in Nigeria. In all countries, over half of stakeholders interviewed were government staff, partner organization staff (e.g. United Nations agency or civil society organization), donor representatives (two countries) or consultant TA providers (see Table S1 for type of stakeholders by country). In addition, two A&T headquarters staff members, three A&T regional staff members and one global-level donor staff member participated in interviews about A&T’s broad TA approach.

| TABLE S1. Summary of key informants interviewed by characteristics | | | | | | |
| --- | --- | --- | --- | --- | --- | --- |
| **Country** | **A&T Staff** | **Government** | **Partner Organization** | **Consultant TA Provider** | **Total** | **Number of women** |
| **Bangladesh** | 4 | 1 | 6 | 1 | **12** | 8 |
| **Burkina Faso** | 4 | 6 | 4 | 0 | **14** | 6 |
| **Ethiopia** | 5 | 4 | 1 | 0 | **10** | 3 |
| **India** | 5 | 2 | 5 | 0 | **12** | 4 |
| **Nigeria** | 4 | 8 | 2 | 1 | **15** | 9 |
| **Vietnam** | 4 | 2 | 4* | 0 | **10** | 6 |
| **Global/Regional** | 5 | — | 1 | 0 | **6** | 4 |
| **TOTAL**  **(% of total)** | **31**  (39%) | **23**  (29%) | **23**  (29%) | **2**  (3%) | **79**  (100%) | **40**  (51%) |

*Includes one stakeholder from a partner organization in Cambodia as part of the regional knowledge exchange.

## Country Summaries of Technical Advisory TA

Boxes A to F provide a summary of each country’s Technical Advisor TA activity included in this assessment, including the country context, background on A&T’s involvement, the main TA activities, results achieved, any specific challenges experienced and the follow-up actions that need to be taken.

| Box A: Bangladesh Professional Medical Associations Support for BMS Act Awareness & Adherence | |
| --- | --- |
| **Context:**  The Bangladesh Breastmilk Substitutes, Infant Foods, Commercially Manufactured Complementary Foods, and the Accessories Thereof (Regulation of Marketing) Act (the Bangladesh BMS Act) was adopted by Parliament in 2013 and supported with additional bylaws in 2017. The Bangladesh BMS Act, based on the Code, was developed specifically to ensure that mothers and families receive accurate and unbiased information about the healthiest way to feed their infants and young children— free of commercial influence. | |
| **Background on A&T’s involvement:** Previously, A&T worked closely with the Bangladesh Paediatric Association (BPA) on IYCF training and SBCC and with the Ob Gyn Society of Bangladesh (OBGSB) on maternal nutrition training and SBCC. In the current phase, one of A&T’s policy and program advocacy priorities is to strengthen the implementation of the BMS Act. Collaboration with professional medical associations has been a key part of this work, as they are very influential in society, both technically and socially. High-level policymakers listen to them. However, not all association members are fully supportive of the BMS Act; there is a wide range of opinions and views that can make this collaboration quite sensitive at times. | |
| **TA Objective:** To provide TA to the executive members of the OGSB and BPA on the importance and benefits of MIYCN based on evidence to raise awareness, foster collaboration, and advocate for BMS Act compliance | |
| **TA Activities:**   1. Signed Letters of Collaboration with OGSB and BPA separately to formalize the partnership for joint advocacy 2. Met with executive members to share the latest MIYCN evidence generated by A&T on BMS Act compliance and best practices for maternal nutrition; developed joint action plan 3. Shared A&T’s existing (Government-approved) tools and materials and built capacity of the executive members on ways to effectively utilize them with its members, affiliated organizations and GoB to enable social and behavior change on key MIYCN practices 4. Jointly developed and disseminated technical briefs on upholding the BMS Act 5. Facilitated joint public webinars and meetings on IYCF and the BMS Act (e.g. National Nutrition Week 2020-22, World Breastfeeding Week 2021), including results from Code violations assessment in urban areas (2018) 6. Involved OGSB and BPA as technical partners in relevant stakeholder and technical consultation meetings | |
| **TA Results:**   1. Endorsement by both OGSB & BPA of BMS Act briefs for 3 target audiences that were co-developed with IPHN (OGSB & BPA logos on briefs) 2. Increased awareness among OGSB & BPA members of the BMS Act and their responsibilities for upholding it. The content of the briefs and meeting discussions have been a ‘wake-up call’ for these medical professionals. 3. Increased prevalence of early initiation and exclusive breastfeeding practices (based on DHS data) 4. Increased engagement by government (IPHN) in advocacy and dissemination of BMS Act assessment results to medical associations; also starting to reach out to other government sectors (e.g. commercial sector, media, Customs, Ministry of Food, Ministry of Disaster) to increase awareness of the BMS Act provisions. | |
| **TA Challenges:**   - Delays due to reluctance by government and some association members to officially endorse the briefs - A&T dependence on the government and other partner organizations to access resources for implementation | **Follow-up Actions Needed:**   - Continued joint advocacy by A&T and the associations with government to take responsibility and leadership role in working together to improve adherence. - Medical associations need to institutionalize BMS Act training in pre-service and in-service education. - Code monitoring assessments every 3-4 years |

| Box B: India Professional Medical Associations Support for IMS Act Awareness & Adherence | |
| --- | --- |
| **Context:** The Infant Milk Substitute (IMS) Act is in place but there is not enough information or awareness about it within the health system. Two different ministries are involved - the Ministry of Women and Child Development (MWCD) is mandated to implement the IMS Act but the Ministry of Health and Family Welfare (MoHFW) is responsible for ensuring IMS Act compliance within its health facilities. There is poor adherence to the IMS Act in the government health system and private health facilities. Adherence is more likely if health care providers have the right information and awareness about the Act but most training packages on IYCF do not touch upon it. In India, a large proportion of pediatricians, gynecologists, general practitioners, and nurses working in both public and private health sector belong to professional medical associations. The associations provide their members access to medical knowledge through evidence-based guidelines, continuous education and capacity building exercises, and networking opportunities. Since 60% of healthcare infrastructure in India is private, professional medical associations offer an opportunity to influence private sector health care providers. | |
| **Background on A&T’s involvement:**  A&T’s partnership with professional medical associations aimed at promoting adoption of strong MIYCN position by the associations, development of evidence-based guidelines on MIYCN for use by their members, sensitization capacity enhancement of evidence-based MIYCN focused clinical practices for mother including adherence to IMS Act and increasing engagement of professional associations in providing continuous support to public health nutrition programs. Although IMS Act monitoring was not a primary focus, all the updated guidance documents, sensitization and capacity building efforts emphasized improving awareness and adherence to IMS Act, with associations taking a strong stand against formula marketing. | |
| **TA Activities:**   1. Signed Letters of Collaboration to formalize arrangement; identified relevant working groups, chapters or committees within each association that could take up specific nutrition issues. 2. **Advocacy** – worked with association leadership to understand and champion MIYCN issues, helping to strengthen this agenda in clinical practice. 3. **Guidelines revision** – created forums that brought all three associations together to jointly review and improve guidelines for consistency across the continuum of care (obstetricians and pediatricians support breastfeeding) 4. **Sensitization and training** – used various platforms to increase association member MIYCN awareness of IMS Act and skills – annual conference sessions, Continuing Medical Education (CME), e-learning courses. 5. **Support for government public health programming** – worked with associations to help take forward the IMS Act, developing a framework for what association could do to incorporate the IMS Act in their training and guidance; organized a broad consultation in 2019 together with the government and Breastfeeding Promotion Network of India to discuss how to improve implementation and monitoring of the IMS Act. | |
| **TA Results:**   - Professional associations adopted evidence-based guidelines to improve MIYCN knowledge and practices. - IMS Act content has now been included in various association sensitization and training efforts, including undergraduate curriculum at some medical colleges and CME programs targeted to association members. - These associations (FOGSI, IAP and IAPSM) are now working together in ways that they never have before – in joint development of updated policy/program guidelines on MIYCN, on integration of MIYCN in pre-service and in-service training, and on assessment of health service provider knowledge and practices.   “This MIYCN is a topic that brought us all together, and there Alive & Thrive India played a very important role also.” (Association member, India)   - Sustained commitment by professional association leadership – identification of MIYCN champions within each association and formation of MIYCN working groups is seen as a model to be replicated for other causes. | |
| **TA Challenges:**   - Took time in some cases to figure out the best subgroup within the association to work with on specific issues - Limited political will among government ministries for IMS Act implementation and monitoring | **Follow-up Actions Needed:**   - IAPSM wants to build on MIYCN implementation research experience and encourage others to design research that helps address ‘real world problems’. - Evaluation of updated guidelines and pre-service/in-service training effects on service delivery quality |

| Box C: Vietnam – SUN Civil Society Alliance Code Monitoring TA | |
| --- | --- |
| **Context:** Vietnam is a regional leader for advancing policies and programs to successfully scale up breastfeeding, having strengthened its national Code of marketing of BMS and national maternity protection. The Nutrition Technical Working Group is composed of CSOs and other development partners who work on nutrition, including WHO and UNICEF. Due to the political situation, CSOs are very careful about how they hold the government accountable for its responsibilities. | |
| **Background on A&T’s involvement:**  The SUN Civil Society Alliance was established in 2019, includes 14 members and is led by various CSOs on a 2-year rotational basis. During the early months of the pandemic, A&T staff identified an increase in the number of Code violations being committed by companies promoting infant formula.^20^ A&T documented these violations and shared them with the Nutrition Technical Working Group members. They drafted a letter and circulated it to the members to sign, with the aim of having a larger number of organizations sign it to put pressure on the Ministry of Health to take action. However, when they circulated the letter for endorsement, they received feedback from SUN CSA members, asking what the Code was about and showing a lack of knowledge. This motivated A&T to organize a training for the SUN CSA member organizations on the Code, the importance of monitoring Code violations and the role of civil society in this area. | |
| **TA Objective:** To strengthen the capacity of SUN CSA members to participate in joint monitoring and advocate together with the government to address Code violations. | |
| **TA Activities:**   1. Conducted training needs assessment for SUN CSA member organizations 2. Engaged with the government health inspectors to co-design and co-facilitate the training workshop – A&T presented defining the violations and explaining it in easy-to-understand language to the member orgs; the health inspectors were in charge of providing a description of the Vietnam context, how they have taken violations into consideration and the mechanism for civil society to report the violations to the government in Vietnam. 3. Provided ‘thought leadership’ to the SUN CSA | |
| **TA Results:**   - Government action taken to address the Code violations reported by SUN CSA; identification of loopholes in the existing legislation that companies are using to get around the Code. - Increased knowledge among SUN CSA member organizations about the Code, how to monitor violations and follow-up with the government on actions taken. This has contributed to increased engagement of members in detecting Code violations as well as other related issues (workplace lactation policy and input on nutrition and the Code for program targeted to ethnic minority population). - Code compliance has been prioritized by members in the SUN CSA workplan for 2021-2023. - Contributed to subsequent TA with the government to develop an application that uses artificial intelligence to detect Code violations. - Increased capacity of SUN CSA members and government to respond to an infant formula safety issue, with health safety inspectors in Vietnam supported by the SUN CSA in Vietnam and Cambodia. | |
| **TA Challenges:**   - Companies are using legal loopholes which limit the government’s ability to act on the Code violations | **Follow-up Actions:**   - Work with the SUN CSA to strengthen the Law on Food Safety that will be reviewed by the government this year. |

| Box D: Burkina Faso – Technical support to revise BMS Code decree | |
| --- | --- |
| **Context:** Burkina Faso first issued a decree to regulate the marketing of breastmilk substitutes (BMS) in 1993. The decree was never enforced and has not been updated with new WHA resolutions since that time. It also had shortcomings related to monitoring, including sanctions. Partners had tried several times since 2002 to revise the decree but were unsuccessful. As a result, there were repeated violations of the Code in the country. | |
| **Background on A&T’s involvement:**  A&T advocated to the MoH and its partners to update the decree for strengthening and enforcing the Code. In 2020, A&T contributed to the process in which the decrees were updated and finalized to include three components: the main decree regulating the marketing of BMS, the second one describing how to and who oversees implementing the norms and standards of BMS packaging, labeling and promotion, and the third that provides guidance on setting up a monitoring committee. The finalization process includes sharing the decrees with all departments of the Government that could be affected directly or indirectly by the updates to the Code. | |
| **TA Activities:**   1. **Advocacy with government and partner organizations to work together to update the decree.** 2. **Alliance building** – A&T negotiated with partner organizations to fund different components of the process, including the national and international legal consultants who worked on the decree. 3. **Roadmap preparation** 4. **Technical and financial support to MOH/Technical Secretariat for Food and Nutrition (STAN) and partners to advocate at the highest level** with the different ministries concerned to explain the need to revise the Code and sign it. 5. **Support to STAN and BMS Code allies in preparing for a technical meeting** to address concerns raised by other Ministries and convince them to keep the articles in the decree strong. 6. **Surveillance of BMS Code violations –** A&T supported the (STAN)/MoH to assess Code violations in health facilities as per the Netcode protocol in November 2021. | |
| **TA Results:**   - Increased awareness of the Code by government and other stakeholders. - Adoption and signing of the new Code decree by all relevant ministries. - MOH has committed to putting in place a plan for scaling up IYCF - Hospital standards for maternal and child health are being revised to take into account the aspects of the Code related to quality of care - Signing of the Code will allow the MOH to accelerate the implementation of BFHI - Complemented with Stronger with Breastmilk Only campaign, this work has contributed to increase in breastfeeding and less visible violations by companies/manufacturers of BMS products. | |
| **TA Challenges:**   - As the number of stakeholders involved increased, so did the number of meetings required to resolve issues raised by various stakeholders - Hiring the government lawyer was a good decision but resulted in longer than expected timelines due to other priorities | **Follow-up Actions Needed:**   - Remaining two Code-related interministerial orders need to be signed - Government needs to act to improve Code enforcement and monitor violations, including information sessions for key stakeholders and establishment of an interministerial committee to coordinate the Code implementation |

| Box E: Ethiopia – Technical Support for revision of BMS directive and sensitization/dissemination | |
| --- | --- |
| **Context:** The Ethiopian government expressed its political aspiration to protect and promote optimal breastfeeding by adopting several generic proclamations and two national implementing directives on the Code of Marketing of BMS supported by regional mechanisms. The government has really taken on the responsibility of improving the national Code and is collaborating with development partners to achieve this goal.^21,22^ | |
| **Background on A&T’s involvement:** A&T has been engaged in efforts to protect and promote breastfeeding in Ethiopia since 2009. In partnership with UNICEF, A&T provides technical and financial support to the Ethiopian Food and Drug Administration (EFDA) and Federal Ministry of Health (FMOH) to advocate for a strengthened version of the Code. A&T also works with the Regional Health Bureaus in six regions of the country. | |
| **TA Objective:**  Adaptation of BMS code through compiling evidence on benefits of the BMS code and developing policy briefs and advocacy packages | |
| **TA Activities:** A&T provided technical and financial support for a series of activities focused on strengthening the Code, sensitizing key stakeholders about the Code’s implications for their respective areas of responsibility, and initiating steps to strengthen the monitoring of Code violations within health facilities. At the regional levek, A&T supported the RHBs and regional regulatory teams.   1. Desk review comparing Ethiopia’s BMS Code against the International Code 2. Technical support for endorsement of new proclamation (1112/2019) 3. Develop, print, and disseminate **posters** on prohibition of BMS advertisement in health facilities, translated in five local languages 4. Technical and financial support (with UNICEF) to the **revision of the BMS directive** to adopt the full provisions of the Code – included translation from English to Amharic by qualified person due to legal language used. 5. Develop a **one-page brief summarizing the Baby Food Control Directive provisions and penalties**, translated into five local languages, printed and disseminated; used during sensitization workshops. 6. Developed **advocacy briefs for health workers, media and policymakers** to sensitize them about the Code 7. Organize **sensitization workshops for wide range of stakeholders at federal and regional levels.** 8. Technical support to develop **monitoring system for government to assess Code violations** at health facilities, including identification of 10 violation-related indicators to include in health inspection checklist. | |
| **TA Results:**   - BMS directive revision and adoption - Code-related materials and tools – one-page brief, advocacy briefs for target groups, posters – produced, translated into local languages and disseminated at national and regional levels - Changes in stakeholder awareness and behaviours – anecdotal evidence of health worker promotion of breastfeeding and media professionals reporting on the benefits of breast milk - Enhanced capacity for monitoring Code violations using health inspection checklist | |
| **TA Challenges:**   - Planned assessment of Code violations in urban areas was canceled due to the civil war - Frequent turnover in government staff - Limited reach of training and sensitization activities to field-level health workers and private sector health care providers | **Follow-up Actions Needed:**   - Regions to adapt the national directive to their local regional directive - EFDA requesting continued support as they implement the Code and start handing out penalties - Need to gather evidence on Code violations in various environments – markets, health centers, etc. |

| Box F: Nigeria – Technical support to develop NAFDAC 5-year strategy | |
| --- | --- |
| **Context:** The National Agency for Food and Drug Administration and Control (NAFDAC) is the food and drug regulatory body in Nigeria, with structures and staff at both federal and state level (36 states). As the regulatory authority for the Code, NAFDAC coordinates all Code activities, monitors Code compliance and applies sanctions when violations are detected, as stipulated in the National Regulations on Marketing of Infant and Young Children Food and Other Designated Products 2019. While six of 11 Technical Directorates are involved with Code implementation, the Food Safety and Applied Nutrition Directorate leads monitoring exercises for compliance. However, work on the Code is just a small aspect of what they do. | |
| **Background on A&T’s involvement:**  As part of efforts to support improved breastfeeding practices, A&T has worked with NAFDAC since 2016 to improve Code implementation, monitoring and compliance. A member of the National Technical Committee on the BMS Code, A&T has provided technical and financial support to Code revisions and support to the government in getting the Code revisions signed into law, including support during key meetings with stakeholders opposed to the regulations. A&T has also supported Code monitoring tools refinement and staff training, as well as Code monitoring efforts in southwest states. | |
| **TA Objective:**  To enhance performance, NAFDAC requires a robust National Strategic Plan to serve as a roadmap for the effective implementation, monitoring and enforcement of the National Regulations on the Marketing of Infant and Young Children Food and Other Designated Products/International Code of Marketing of Breast-milk Substitutes. A structured Plan will facilitate sustainable and consistent Code implementation in Nigeria. | |
| **TA Activities:**  The National Strategic Plan was prepared under the guidance and leadership of NAFDAC and Code experts at national and international levels, with support from A&T and other Development Partners. The strategy development process involved the following steps:   1. Concept note, advocacy and consensus building with NAFDAC leadership 2. Co-development of TOR with NAFDAC 3. Selection and hiring of consultant to conduct desk review and draft strategy (consultant worked from June-August’2020) 4. **Draft strategy developed** – steps included document review for situation analysis on the Code, development of implementation framework and circulation of draft document to all key stakeholders, presentation of draft document to the Technical Committee, finalization and submission of the draft document 5. Review and validation of draft strategy with all stakeholders 6. **Development of costing and monitoring framework** with NAFDAC and development partners 7. A&T and UNICEF supported participation of NAFDAC at the regional commemoration of the Code on May 26, 2021. NAFDAC shared Nigeria’s experiences and lessons learnt on monitoring and enforcement of the Code at the event. 8. **Launch of NAFDAC strategy** in association with commemoration of 40th anniversary of the Code (August 2021) | |
| **TA Results:**   - NAFDAC’s five-year costed strategy was launched in August 2021 - A&T and UNICEF’s support has contributed to strengthening NAFDAC’s enforcement of the Code through having a better articulated operational and policy framework (national regulations), an active Technical Committee on the Code, and awareness creation efforts. | |
| **TA Challenges:**   - Changes in NAFDAC leadership caused delays and required renewed advocacy efforts by A&T - Efforts to cost the strategy were delayed | **Follow-up Actions Needed:**   - NAFDAC needs to put the strategy into operation, including sufficient human and financial resources - Need for states to adapt the national strategy, set up state BMS Committees and carry out sensitization and training activities - Build government and partner stakeholder capacity to conduct monitoring & assessment of Code violations |

## Country Summaries of Capacity Development for Maternal Nutrition TA Activities

| Box H: Bangladesh Urban Implementation Research to Strengthen MIYCN Counseling | |
| --- | --- |
| **Context:** Urban health service delivery is quite fragmented in Bangladesh, with a mix of NGO platforms and the government’s Urban Primary Healthcare Project (UPHCSTB), under the Ministry of Local Government. Significant gaps in access to quality nutrition services exist; in particular, there is low coverage of quality MIYCN counseling during ANC and sick child visits. There is also low demand for these services due to lack of awareness of their importance.^23^ | |
| **Background on A&T’s involvement:** Much of A&T’s work to date in Bangladesh has focused on strengthening nutrition service delivery in rural areas. However, the training materials, job aids and SBCC approaches developed could be adapted to fit the urban context. Building on formative research conducted with iccdr’b in 2019, A&T designed an implementation model for delivering quality and standard MIYCN counseling services in urban MNCH services settings which will serve as a model to be incorporated into the existing urban health service delivery package. A&T conducted implementation research to test the feasibility and outcomes of the approach. | |
| **TA Objective:** To build the capacity of two NGOs hired as implementation partners to strengthen MIYCN service delivery in urban facilities and generate demand for those services, as well as advocate with GOB and partners for inclusion of learnings into urban strategy and project design. | |
| **TA Activities:**   1. Development of urban MIYCN counseling implementation guide, training materials, SBCC materials, job aids, supportive supervision tools (e.g. checklist) and monitoring tools (Excel tool) 2. Build capacity of project managers from two NGOs (Radda MCH-FP Center and Marie Stopes Bangladesh) who operate urban health facilities, training them how to implement MIYCN counseling model, including supportive supervision and strategic use of monitoring data 3. Build capacity of MIYCN Counselors and Community Workers on MIYCN and job responsibilities, including counseling skills and use of SBCC materials developed for this context 4. Technical support, ongoing training and mentoring to strengthen quality of implementation 5. Adaptation due to COVID-19 pandemic restrictions, including remote training, supervision, mobile-MIYCN 6. Advocacy with GOB and partners to include this model in urban strategy and project design. | |
| **TA Results:**   1. Program package to support implementation, including operational guide, training materials, SBCC materials, job aids and supportive supervision tools 2. Increased capacity of the two NGOs and their staff to provide quality MIYCN counseling services 3. Increased demand for nutrition counseling services among women/mothers of young children 4. NGO expressed commitment to continue providing nutrition counseling using this model with cost-recovery 5. Government (IPHN) expressed commitment to adopt and scale-up the model, including set-up of the recommended counseling room in the new IPHN building 6. Evidence generated on the feasibility of implementing this approach and outcomes associated with it (pending publication of evaluation findings) | |
| **TA Challenges:**   - Inability to conduct research using government platform due to contractual issues - COVID-19 pandemic onset during first week of training; required multiple adaptations to approach and limited dissemination of baseline findings to government and other stakeholders - High levels of facility staff turnover | **Follow-up Actions Needed:**   - Share endline survey results with stakeholders - NGOs to sustain implementation using cost-recovery model - IPHN, UPHCSTB and NNS to consider how best to scale-up the approach in both urban and rural contexts |

| Box I: Burkina Faso Integration of Maternal Nutrition Interventions in Maternal Health Services | |
| --- | --- |
| **Context:** There is a strong enabling environment for nutrition in Burkina Faso. The MoH adopted the 2016 WHO guidelines on ANC in 2017 and the following year developed ANC directives, in partnership with WHO and other partners like A&T. However, there was a need to operationalize the revised ANC guidelines and test their feasibility. Development partners, including UNICEF and WHO, were also interested to support the government in testing and scaling up effective maternal nutrition interventions. | |
| **Background on A&T’s involvement:** A&T worked with two divisions in the MoH – *la Direction de la Sante et de la Famille* (DSF) and *la Direction de la Nutrition* (DN) – to design and implement a maternal nutrition implementation research project in four health districts in two regions (Mar’2020-Jan’2021). The study tested the feasibility of integrating a package of nutrition interventions as part of the ANC platform in these districts (40 health facilities). The interventions included strengthening iron-folic acid (IFA) delivery and consumption, dietary diversity counseling, weight gain monitoring, as well as EIBF and EBF promotion. A&T also supported the MOH in strengthening facility-community level linkages and increasing demand for these services among women and their household members. IFPRI conducted the evaluation through two cross-sectional surveys (Dec 2019, Feb 2021). | |
| **TA Objective:** To integrate maternal nutrition interventions in maternal health services at scale following results of the implementation research. | |
| **TA Activities:**  develop and validate maternal nutrition training modules, package of communication tools for various levels, national monitoring & supervision tools to monitor the coverage and quality of maternal health services   1. Support to DSF and DN to disseminate the maternal nutrition implementation results with key stakeholders and incorporate learnings into upcoming research projects by WHO (ANC) and UNICEF (MMS). 2. Support to MOH to revise and validate the national nutrition training modules based on evidence and lessons learned from A&T’s research and the government’s recent adoption of MMS. 3. Support two regions to scale-up implementation of maternal nutrition interventions during ANC, including addition of one day training on maternal nutrition for health care providers and community health workers into other related training (e.g. essential newborn care). 4. Provide national training of trainers on the maternal nutrition package in collaboration with UNICEF 5. Support to the government to finalize the national ANC guidelines 6. Support to MOH to develop a national scale-up plan, based on the lessons learned, recommended good practices and feasible (simplified) maternal nutrition intervention package of interventions. | |
| **TA Results:**   1. MOH updated and validated **national maternal nutrition training modules** 2. Scale-up of implementation of minimum package of maternal nutrition interventions across all health facilities in the four districts where the research study was done. 3. Integration of maternal nutrition interventions in region-level reproductive health supervision checklist. 4. MOH standards on quality of maternal and neonatal care are now integrated, building on the learning from the maternal nutrition research. 5. Enhanced capacity of health care providers to monitor weight gain during pregnancy and counsel pregnant women accordingly, based on the new tools developed during the research. | |
| **TA Challenges:**   - High cost for government to print job aids and SBCC materials (limits scale-up of these tools) - Coordination with other development partners to use MOH-endorsed maternal nutrition training modules | **Follow-up Actions Needed:**   - Advocacy for government commitment to scale-up the simplified package of maternal nutrition interventions - Lower cost reproduction of posters, job aids and SBCC materials |

| Box J: India – Supporting scale-up of maternal nutrition services within the health system | |
| --- | --- |
| **Context:** The government of India recognizes the importance of addressing maternal nutrition in its efforts to improve maternal and child survival and nutritional outcomes. With suboptimal coverage of both ANC and nutrition services, there is a need to integrate maternal nutrition within existing platforms while also strengthening health service delivery systems. Two government bodies are responsible for delivering these actions – micronutrient supplementation, deworming, health check-ups, and curative interventions are delivered through the National Health Mission (managed by the MoHFW) and food supplementation and growth monitoring are delivered through the Integrated Child Development Scheme (ICDS, managed by the Ministry of Women and Child Development, MWCD). Development partners engaged in maternal nutrition include UNICEF, UP-TSU (managed by India Health Action Trust) and Nutrition International. | |
| **Background on A&T’s involvement:**  A&T India prioritized maternal nutrition from the outset. In 2016, building on evidence generated by A&T in Bangladesh on integrating maternal nutrition in ANC services and the release of WHO’s ANC guidelines, A&T organized a roundtable at the national level to raise maternal nutrition on the agenda and convince the government to take action. The MoHFW agreed to work on the issue but wanted evidence of what works in India’s health system. A&T designed and conducted an implementation research study in UP (2018-2019) to test a minimum package of nutrition interventions as part of the outreach platform for routine ANC. At the same time, the government convened a technical expert committee to review the national ANC guidelines and develop comprehensive operational guidelines to address maternal nutrition. | |
| **TA Objective:** To provide technical support for government and partner organization efforts in UP, Bihar and Jharkhand states to integrate and strengthen maternal nutrition interventions during ANC, and national level technical support to MoHFW to strengthen the institutional enabling environment for maternal nutrition services. | |
| **TA Activities:**   1. **Dissemination of implementation research findings** at both state and national levels. 2. Development of a **maternal nutrition operational guide** targeted to block, district, and state health and ICDS officials to assist them in planning, managing and implementing maternal nutrition programs 3. **Technical support to partner organizations** in different states to integrate maternal nutrition, utilizing the guidelines, training modules for frontline workers and supervisors, and job aids/tools co-developed with the government as part of the implementation research:    - UP-TSU to support state government to scale-up maternal nutrition training across the districts    - UNICEF Bihar to organize district wide training for integrating maternal nutrition    - UNICEF Jharkhand to help government integrate maternal nutrition protocols in their ANC SOP 4. **Technical support to MoHFW** at national level to develop program guidelines and protocols for integrating maternal nutrition in ANC. 5. **Maternal Nutrition Partners Consortium** organized Maternal Nutrition e-Dialogues, regular technical webinar series on topics (e.g. maternal obesity, thinness, anemia), hosted by National Institute of Nutrition, along with FOGSI and supported by UNICEF, A&T and others. 6. **Advocacy** at national level for administrative mechanisms and approval of state planning and budgets for maternal nutrition activities 7. **Sensitization and training webinars** with FOGSI and in medical colleges for integrating maternal nutrition in health facilities. | |
| **TA Results:**   1. Increased recognition by government and development partners that maternal nutrition is a priority for action. 2. Strengthened health system in the UP districts where the IR was conducted – the process has improved and people are working, providing services. 3. Strengthened collaboration with UP state government and partners (UP-TSU) based on practical tools and learning supported by A&T 4. Increased capacity of UP-TSU to support government in training frontline workers on maternal nutrition, including technical knowledge, training materials and budget. 5. Commitment by ICDS in UP State to scale-up across all 75 districts of mobile phone application that strengthens supportive supervision and strategic use of data components (another A&T TA activity that is ongoing). 6. Acceptance by state governments (e.g. Bihar) to integrate maternal nutrition into existing program modalities 7. Inclusion of maternal nutrition training in state PIPs, resulting in increased funding available for nutrition actions and increased motivation for the government to implement these. 8. Increased capacity by government staff to use monitoring data to inform program strengthening. 9. Inclusion of nutrition screening in national government guideline for high risk pregnancies. (TBC) | |
| **TA Challenges:**   - COVID-related impact on results dissemination activities - Need to establish new government mechanisms for planning and budgeting of maternal nutrition actions - Shifts in political leadership and delays in finalizing the revised national ANC guidelines - Need for health system strengthening as well as technical nutrition intervention strengthening | **Follow-up Actions Needed:**   - Ongoing A&T technical support to government and partner organizations for training, monitoring & evaluation, |

Note: A&T had developed an implementation manual, training modules for frontline workers and supervisors and job aids /tools as part of the implementation research in consultation with state government.

## Country Summaries of Capacity Development for Strategic Use of Data TA Activities

| Box K: Ethiopia | |
| --- | --- |
| **Context:** Although the National Nutrition Program II calls for coordinated, multi-sectoral actions, it has been challenging for the government to set up an integrated nutrition information system. A multisectoral nutrition scorecard has been developed and seeks to bring information together from the various sectors. The health sector’s HMIS is well-established in Ethiopia but its quality and utility for nutrition program monitoring and improvement is limited, in part because of a historical major emphasis on screening and treatment of acute malnutrition. | |
| **Background on A&T’s involvement:** In its second phase of work in Ethiopia (2018-2022), A&T has focused on building capacity of FMOH and Regional Health Bureaus (RHBs) in six regions to support woredas in planning, budgeting, and implementing actions to achieve MIYCN targets in NNP II. The need to strengthen information systems for nutrition was identified during the regional organizational capacity assessments conducted. **Improving data quality and utilization was one of the objectives in A&T’s project agreement.** In four of the six regions, A&T included an M&E Advisor as one of the program staff embedded in the RHB team. | |
| **TA Objective:** To improve nutrition (particularly MIYCN) data use at federal and regional levels. | |
| **TA Activities:**   1. Hold training workshops on DHIS2 for regional M&E officers and nutrition officers to strengthen mutual understanding of nutrition indicators in the DHIS2. 2. Conduct assessment on the various information systems that capture nutritional data (especially those that pertain to MIYCN) in four NNP II signatory sectors. (hired consultancy firm to support the assessment) 3. Disseminate the results of the assessment to stakeholders at both federal and regional levels, discuss priorities for action and identify where A&T can support. 4. Work with RHB to develop TOR for performance management team (PMT) teams to revitalize regular meetings and strengthen nutrition information use (specifically MIYCN) culture at directorate and case team level; provide technical support to nutrition case teams in quarterly nutrition data analysis and use. 5. Advocate for and support RHBs to include nutrition indicators in Routine Data Quality Assessment (RDQA) and conduct RDQA in sampled health facilities. 6. Inclusion of MIYCN indicators in RHB supportive supervision checklist | |
| **TA Results:**   1. Report on Landscape Analysis of Nutrition Information Systems in Ethiopia, by Telos Consulting, Jan’2020 2. Revitalized performance management team meetings in the RMNCH directorate in three regions 3. Enhanced capacity of the nutrition case team to conduct weekly, monthly and quarterly reviews of data reported in DHIS2 and increase feedback to lower levels on data quality and performance 4. Integration of nutrition in health sector tools (e.g. nutrition indicators in supportive supervision checklist) and information systems content in nutrition implementation guide and training materials. (Oromia RHB) | |
| **TA Challenges:**   - Delay in assessment report dissemination due to COVID-19 pandemic restrictions; disruption in TA for two regions due to conflict. - Although the project sought to strengthen multi-sectoral nutrition programming, resource constraints required A&T to narrow the scope of work on NIS to the health sector. - Large regions require large-scale efforts that go beyond what government or A&T can support. | **Follow-up Actions Needed:**   - Need for sustained training and supportive supervision at lower levels to improve data quality for nutrition services. - Advocacy with RHBs to include nutrition indicators in RDQA on routine basis. |

| Box L: Nigeria - building capacity in Kaduna State for IYCF data quality and use | |
| --- | --- |
| **Context:** Improving IYCF practices is a high priority for the government and its development partners. At the federal level, the FMOH M&E Technical Working Group successfully advocated for inclusion of five nutrition indicators that were heavily focused on IYCF in the NHMIS. The national tools were piloted by the FMoH with support from partners (including A&T) and finalized in 2019. With the revised NHMIS tools, data on IYCN is now readily available on the national data platform (DHIS2). In Kaduna State, there is a clear demand by the highest level of government for data to be used in decision making. | |
| **Background on A&T’s involvement:** As part of A&T’s efforts to improve IYCF practices in Nigeria, they have worked closely with the FMoH and stakeholders in Kaduna and Lagos states to strengthen information systems for nutrition. A&T was a key player in the FMoH M&E Technical Working Group. In addition to supporting the rollout of the 2019 NHMIS tools, A&T also supported health facilities in Lagos and Kaduna states to migrate from Excel-based reporting to the DHIS2 through mobile app reporting. A&T has also worked with community health workers and private sector health facilities to record data and include it in the health facility data reporting system. | |
| **TA Objective:** To jointly work with Kaduna State Primary Healthcare Development Agency (KDSPHCDA) to build capacity and develop a monitoring framework for use in monitoring and mentoring of IYCF data in the state. | |
| **TA Activities:** Building on earlier work to include key IYCF indicators in the NHMIS, A&T has continued to work with Kaduna State stakeholders to strengthen both health facility and community-level routine information systems. In addition to developing project-specific tools to improve data entry and reporting on IYCF indicators, A&T supported training and joint supportive supervision for enhanced capacity of state and LGA-level health workers to record, review for quality and report data up the chain.   1. Ensure all 2019 NHMIS tools are available and in use, especially the GMP register 2. Conduct joint supportive supervision (data quality assurance) visits with LGA M&E officers to PHCs and private health facilities to build capacity of staff in data validation and troubleshooting data errors 3. Develop a monitoring and mentoring framework in close collaboration with KDSPHCDA 4. Participate in state government data review meetings | |
| **TA Results:**   1. Enhanced knowledge and skills of state and LGA-level health workers to use 2019 NHMIS tools, including electronic data entry to DHIS2 using mobile phones 2. Increased availability of data tools (registers, summary forms) at health facilities 3. Increased quality of data reported – timeliness, high reporting rate (80-90%), fewer data gaps, improved internal consistency 4. Integration of nutrition indicators in routine monthly and quarterly data quality review meetings | |
| **TA Challenges:**   - High rate of staff turnover, staff transfers that resulted in loss of trained personnel - COVID-related restrictions resulted in low and incomplete data documentation and reporting - State reliance on A&T technical support (and presence) to implement monitoring framework - Mobile app dependent on funding for network access and regular maintenance of phones | **Follow-up Actions Needed:**   - Strengthen use of data through enhanced analytics, data visualization and use of the dashboard - Increase capacity of nutrition focal persons to access and use NHMIS data to inform program decisions - Advocate for government funding to sustain network access for mobile phones using DHIS2 app |

| Box M: Vietnam – COE facility checklists and mothers’ experience survey as monitoring tool | |
| --- | --- |
| **Context:** In 2018, WHO/UNICEF provided updated global guidelines for the Baby Friendly Hospital Initiative and issued a call to countries to better integrate BFHI in existing healthcare systems to increase sustainability and coverage. The government had implemented BFHI in Vietnam but coverage was very low – only 0.4% of mothers delivered their baby in a BFHI-accredited hospital. The MOH recognized the problem and was willing to act. At the same time, hospitals in Vietnam that provide maternity services were facing increased competition with private hospitals for customers. So they were keen to build a good reputation that enabled them to attract more clients. | |
| **Background on A&T’s involvement:** With funding from Irish Aid, A&T works to strengthen the capacity of the health system in Vietnam to deliver high-quality breastfeeding-friendly services to mothers and infants. Building on its experience in promotion of breastfeeding and essential newborn care in Vietnam as well as its strong working relationship with the Maternal and Child Health department (MOH), A&T conceptualized the Center of Excellence (COE) for Breastfeeding initiative. A&T also partnered with Da Nang Hospital for Women and Children’s Learning & Research Center (LRC) for Newborn Care and Human Milk to provide clinical expertise as master trainers. | |
| **TA Objective:** To develop **checklists for facility assessment, questionnaires and platform for mothers’ experience survey for the Center of Excellence for Breastfeeding** initiative | |
| **TA Activities:**   1. Work with MOH to develop COE designation, evaluation criteria and designation process, with criteria that complement the National Hospital Quality Standards (NHQS). 2. Develop tools and materials to support hospitals to meet the COE criteria (in collaboration with MCH department and Da Nang LRC), including tools to assess and monitor progress (communication products, coaching material, tools, checklists). 3. Invite hospitals (2-3 per province) to join the initiative and provide TA to improve their breastfeeding support practices in collaboration with Da Nang LRC as master trainers and coaches. Build a resource pool of EENC and breastfeeding experts (master trainers). 4. Develop mother experience survey to provide feedback to hospitals on their performance; build capacity of CDC/DOH/MOH to conduct surveys on quarterly basis as independent assessment. 5. Support hospitals who join the COE to identify gaps in practice and develop action plan for improvement. Provide expert observation, coaching and supportive supervision to enable hospitals to reach the benchmarks. 6. Support MOH to create a monitoring process and tool for COE-designated hospitals to continue conducting mother experience surveys on a quarterly basis, as a way to ensure they remain COE compliant. | |
| **TA Results:**   1. In 2022, 70 hospitals across 14 provinces are enrolled in the COE initiative (up from 28 hospitals in 8 provinces in 2019). 23 hospitals are officially designated as COE and 10 are waiting a decision. In Q3 of 2022, 15 of 23 COE hospitals are conducting the quarterly survey; others supported by provincial DOH/CDC. (source: A&T staff) 2. MOH adopted Decision No. 3451 (2019) approving the COE designation criteria and mechanism in obstetric hospitals, along with eight checklists for clinical observation of EENC and breastfeeding; this was updated in 2021 (decision 5913/QD-BYT) to allow pediatric hospitals to participate in the initiative. 3. Enhanced capacity of MOH/DOH and hospitals to obtain regular feedback on hospital EENC practices, with quarterly monitoring surveys of mothers’ experience providing timely data for action. 4. Enhanced knowledge and skills of hospital staff, resulting in improved EENC practices and labor/delivery practices which in turn contribute to better patient outcomes and cost savings for the hospitals^24^ 5. Increased early and exclusive breastfeeding practices reported by women who delivered in COE-enrolled^25^ and designated hospitals^24^ | |
| **TA Challenges:**   - COVID-19 pandemic shifted priorities for hospitals and delayed scale-up process - Low commitment hospital leadership (small number of hospitals) - Capacity of hospitals and DOH to conduct quarterly monitoring surveys | **Follow-up Actions Needed:**   - Identification and capacity building for master trainers and coaches in more provinces. - Build capacity of MOH to take on the coordination role that A&T currently plays and fully institutionalize the initiative |
